# Optimising prehospital trauma triage for older adults: challenges, limitations, and future directions

**DOI:** 10.3389/fmed.2025.1569891

**Published:** 2025-04-22

**Authors:** Naif Harthi, Steve Goodacre, Fiona C. Sampson

**Affiliations:** ^1^Emergency Medical Services Programme, Department of Nursing, College of Nursing and Health Sciences, Jazan University, Jazan, Saudi Arabia; ^2^Sheffield Centre for Health and Related Research (SCHARR), University of Sheffield, Sheffield, United Kingdom

**Keywords:** triage, ageing impacts, trauma, prehospital care, older people

## Abstract

The ageing population presents significant challenges for prehospital trauma care, with older adults experiencing higher rates of undertriage and overtriage due to age-related physiological changes, frailty, and polypharmacy. Standard trauma triage tools, primarily designed for younger populations, often fail to accurately assess injury severity in older adults, leading to delays in definitive care or unnecessary resource use. This narrative review synthesises current evidence on the limitations of existing trauma triage tools for older adults, highlighting challenges such as inconsistent implementation, paramedic training gaps, and age-related biases. The review explores the role of adjusted systolic blood pressure thresholds, frailty assessments, and geriatric-specific triage protocols in improving triage accuracy. While these modifications show promise, their integration into prehospital care remains limited due to logistical and clinical barriers. Key findings suggest that incorporating frailty assessments, refining age-specific triage criteria, and enhancing paramedic education can improve the precision of prehospital trauma triage for older adults. However, significant research gaps remain, including the need for large-scale prospective studies on geriatric-specific triage tools and investigations into the impact of triage modifications on long-term patient outcomes. Standardising geriatric triage protocols, leveraging digital decision-support tools, and addressing disparities in trauma centre access are critical to optimising prehospital care for older trauma patients. Future research should focus on refining triage strategies to enhance decision-making and ensure that older adults receive timely, appropriate trauma care, ultimately reducing preventable morbidity and improving patient outcomes.

## 1 Introduction

The global ageing population presents unprecedented challenges for trauma care systems worldwide (1). Advances in healthcare, lifestyle, and living conditions have extended life expectancy but also increased major trauma among older adults, straining emergency medical services (EMS) (2–5). By 2050, the number of older adults is projected to reach 2 billion globally, according to World Health Organisation statistics (6). Demographic studies further predict that by 2030, 20% of the United States population will be over 65 years of age, with similar trends anticipated in other regions, including the United Kingdom (23% by 2035), Europe (30% by 2050), and Australia (21% by 2054) (3, 7–9). These demographic changes require a reassessment of prehospital trauma care, particularly the effectiveness of triage in identifying and managing major trauma in older adults. Triage is a fundamental component of trauma care, allowing EMS providers to prioritise patients based on injury severity and ensure timely transport to appropriate healthcare facilities (10). However, triage errors remain a concern, particularly in older adults, who may present with atypical physiological responses to trauma (11–13).

Two key challenges in trauma triage are undertriage and overtriage. Undertriage occurs when older patients with severe injuries are transported to a lower level of care, resulting in delays in definitive treatment and poorer clinical outcomes (14–17). While overtriage happens when older patients with minor injuries are taken to major trauma centres (MTCs), placing a strain on resources and increasing healthcare costs (18). These challenges are amplified in older trauma patients due to age-related physiological changes, frailty, and polypharmacy (19). Standard trauma triage tools, designed for younger populations, often fail to accurately identify major trauma in older adults or address their specific needs (20–22). This narrative review synthesises current evidence on the practical challenges and gaps in geriatric-specific trauma triage within prehospital settings. This review seeks to inform the development of tailored and effective strategies for improving trauma triage in older adults, enhancing the identification of major trauma cases.

## 2 Methods

This narrative review synthesises current literature, relying on the methodology outlined in the following subsections.

### 2.1 Sources of information

The review utilised PubMed and Google Scholar, chosen for their comprehensive coverage of biomedical and health sciences research, as well as their multidisciplinary scope.

### 2.2 Keywords and search strategy

The literature search was conducted using keywords and phrases such as “triage”, “trauma”, “older adults”, and “prehospital”. Articles were selected based on their relevance to prehospital trauma triage in older adults. Boolean operators (e.g., AND, OR) were used to combine search terms with synonyms and refine the search results.

### 2.3 Time frame and language restrictions

Studies published between 2005 and 2025 were included to ensure the selection of the most relevant and up-to-date research. Only English-language publications were considered to maintain consistency, clarity, and alignment with the authors’ language proficiency.

### 2.4 Types of literature

The review encompassed relevant peer-reviewed journal articles, systematic and literature reviews, and government reports and guidelines that provide published insights on prehospital triage for older patients with major injuries.

### 2.5 Screening process

Initially, papers were screened based on their titles and abstracts to exclude irrelevant studies. Full-text reviews were then conducted, followed by an assessment of the reference lists of these articles to determine their eligibility concerning the review objectives, resulting in the inclusion of 61 papers. Of these, 28 originated from the United States (1, 4, 20–45), 18 from the United Kingdom (11, 19, 46–61), five from Australia (17, 62–65), three from Saudi Arabia (66–68), two from Germany (69, 70), and one each from Canada (71), Ireland (72), France (73), Belgium (74), Switzerland (75), and Norway (76).

### 2.6 Integration and analysis

The included studies were thematically analysed to identify challenges in practice and gaps in the existing evidence on prehospital trauma triage for older adults. Key themes were developed iteratively, guided by the overarching aim of improving prehospital trauma triage for this population.

### 2.7 Synthesis of findings

The findings were synthesised into a narrative format, aligning with the review’s objectives to identify challenges in prehospital trauma triage for older adults, highlight gaps in current evidence, and explore best practices. The synthesis also involved comparing international approaches to prehospital triage and proposing evidence-based recommendations to enhance triage accuracy and patient outcomes in older trauma populations.

### 2.8 Peer review

The data were reviewed internally by the co-authors and both internally and externally by academics with interests and expertise in emergency care. This peer-review process ensured the accuracy, clarity, and relevance of the findings, which were refined to align with current evidence and best practices.

### 2.9 Data and reference management

Mendeley literature management software was used to systematically document and organise detailed records of the search results, screening processes, and references.

## 3 Age cut-off for defining older patients to developing triage tools

The increasing number of injuries among older patients, coupled with the growing burden on trauma centres, has led to the development of treatment protocols and guidelines tailored to meet the needs of this demographic (1). However, there is no universally agreed-upon definition of what constitutes an ‘older patient’, with thresholds varying across studies, typically ranging from 55 to 65 years or older (41). This variability creates ambiguity about which patients should be managed with geriatric-specific protocols and complicates efforts to standardise trauma triage practices (4, 41). Many studies employ arbitrary age cut-offs when assessing mortality and outcomes in injured older patients, resulting in diverse findings (41). For this review, we acknowledge this lack of consensus but align with the most commonly used definition, considering individuals aged 65 years and older as “older patients”. Where studies use alternative age thresholds, such as 55 years, this is explicitly stated to maintain clarity. This approach aims to balance consistency within the review while respecting the variability present in the existing literature.

Studies have sought to determine the most appropriate age threshold for older patients. For instance, Fakhry et al. examined different age groups to evaluate the relationship between age and trauma outcomes (41). In this multicentre retrospective study in Tennessee, USA, involving over 255,000 patients, mortality rates significantly increased at ages 55, 77, and 82 compared to those younger than 55 years (41). Based on these findings, Fakhry et al. proposed using 55 years as the threshold for defining older patients with injuries, particularly in research focused on mortality and morbidity risks (41). This inconsistency in age thresholds complicates comparisons between studies, impedes the development of standardised trauma triage protocols, and leaves clinicians uncertain about which age group is at greater risk of mortality (1, 41). Future research should aim to establish a more uniform age threshold, enabling better comparability of outcomes and the development of evidence-based geriatric trauma care guidelines.

## 4 Triage process and limitations to identify older adults with major trauma

### 4.1 Traditional trauma triage tools

Trauma triage tools are utilised by paramedics on scene to identify individuals with major injuries (19). These tools generally determine the transport decisions for trauma patients based on the severity of their injuries (42). The triage of injured patients in prehospital settings is crucial for ensuring that those with severe injuries receive care at high-resource hospitals, such as MTCs, while optimising the use of limited trauma resources (43). Since most severely injured patients receive acute care through the EMS, having an accurate and effective prehospital system for appropriately matching patients to hospital resources is a critical component of trauma care (43). An effective trauma triage tool should possess sufficient sensitivity to identify major trauma cases for referral to a MTC of level I/II (similar to the UK’s MTCs or an Australian Major Trauma Service (MTS)) while maintaining specificity to ensure those with non-major trauma can be treated at a lower-level or non-MTC facility (76). Effective triage is fundamental in delivering high-quality care and reducing mortality rates among injured patients (73). It is challenging to estimate the total number of triage tools as they vary by country and even within regions of the same country (11). Common prehospital triage tools, as identified in the included papers, include the National Field Triage Decision Scheme and the Ohio Trauma Triage Protocol, with most studies focusing on these North American tools (Table 1).

**Table 1:** A summary of traditional prehospital trauma triage tools for older adults: features, evidence, and limitations.

Triage tool / protocol	Key features	Supporting evidence	Limitations/ gaps
US field triage decision scheme	Four-step triage process: 1. Physiological: GCS < 13, SBP < 90 mmHg, abnormal respiratory rate; 2. Anatomical: penetrating injuries, long-bone fractures, skull/pelvic fractures; 3. Mechanism of Injury: falls >20 ft., vehicle ejection, pedestrian struck; 4. Special Considerations: age ≥55, anticoagulant use, pregnancy, clinical judgement Designed to optimise use of trauma system resources	Evaluated across 94 EMS agencies and 122 hospitals Sensitivity: 85.8% (overall), 79.9% in older adults Specificity: 68.7% Final triage including hospital input improved sensitivity to 82.6%	Lower sensitivity in older adults, raising undertriage risk Steps 1 and 2: high specificity but lower sensitivity Steps 3 and 4: improve sensitivity but increase overtriage Lacks frailty or comorbidity assessment No standardised geriatric-specific modifications implemented
Ohio trauma triage protocol (incl. geriatric-specific criteria)	Three main components: 1. Physiological: GCS ≤ 13, SBP < 90 mmHg, respiratory rate < 10 or >29, loss of consciousness >5 min; 2. Anatomical: penetrating injuries, flail chest, spinal cord injury, pelvic fractures, amputations, burns >10% TBSA; 3. Additional Considerations: mechanism of injury, age, comorbidities Geriatric-specific adjustments (2009): Raised SBP threshold to <100 mmHg GCS ≤ 14 for TBI Single long-bone fracture Standing falls with TBI Multiple body region injuries	Analysed data from 101,577 patients (33% geriatric) Geriatric criteria sensitivity: 93% (vs. 61% adult criteria) Specificity: 49% (vs. 61%) Improved detection of ICU admission, mortality, and surgery needs in older adults	Reduced specificity leads to increased overtriage May strain trauma centres due to more non-critical patients being referred Developed for Ohio—limited generalisability to other regions Does not include frailty screening despite age-adjusted criteria Requires further validation in diverse EMS systems

#### 4.1.1 The United States US field triage decision scheme

The US Field Triage Decision Scheme, established by the American College of Surgeons Committee on Trauma (ACSCOT) over two decades ago, has undergone periodic revisions and updates (43, 44). Updated in 2011 by the Centres for Disease Control and Prevention (CDC) in collaboration with the National Expert Panel on Field Triage, the scheme refines the criteria for identifying patients who would benefit from specialised trauma centre care (45). It serves as a decision-making tool for EMS personnel, ensuring that trauma system resources are effectively utilised while minimising the risk of under-triage (45). The triage process consists of four sequential steps as described below by McCoy and colleagues (45):

Step one: Physiological Criteria involves a rapid assessment of vital signs and consciousness levels to identify critically injured patients. Key indicators include a Glasgow Coma Scale (GCS) score of less than 13, a systolic blood pressure (SBP) of less than 90 mmHg, and an abnormal respiratory rate (fewer than 10 or more than 29 breaths per minute, or fewer than 20 in infants under 1 year old). Additionally, patients requiring ventilatory support are classified as high risk and should be transported to a facility that provides the highest level of trauma care. Step two: Anatomical Criteria focuses on injuries that may necessitate specialised care even if the patient’s vital signs initially appear stable. This includes penetrating injuries to the head, neck, torso, or proximal extremities; chest wall instability or deformity (such as flail chest); two or more proximal long-bone fractures; crushed, degloved, mangled, or pulseless extremities; amputations proximal to the wrist or ankle; pelvic fractures; open or depressed skull fractures; and paralysis. Patients meeting these criteria should be transported to the highest level of care within the trauma system.

Step three: Mechanism of injury considers the nature and force of the trauma, recognising that some injuries may not be immediately apparent but could still be severe. High-risk mechanisms include falls greater than 20 feet for adults or 10 feet (or two to three times the child’s height) for children. Severe road traffic collisions, such as those involving significant intrusion, ejection, or a fatality in the same passenger compartment, also warrant concern. Similarly, incidents involving pedestrians, cyclists, or motorcyclists struck at high speeds may indicate severe underlying trauma, necessitating transport to a trauma centre. Step four: Special Considerations accounts for patient-specific factors that may increase the risk of severe injury. Older adults, particularly those aged 55 and above, may experience more severe consequences from trauma, with SBP levels below 110 mmHg potentially indicating shock. Paediatric patients should be triaged preferentially to trauma centres equipped for children. Additionally, patients on anticoagulants or with bleeding disorders who sustain head injuries are at high risk for rapid deterioration. Other considerations include burns, pregnancy beyond 20 weeks, and cases where EMS providers use clinical judgement to determine the need for trauma centre care.

Newgard and colleagues evaluated the reliability of the US Field Triage Decision Scheme in identifying major trauma patients (ISS ≥ 16) across 94 EMS agencies and 122 hospitals in the Western United States (43). This scheme demonstrated 85.8% sensitivity (95% CI: 85.0–86.6%) and 68.7% specificity (95% CI: 68.4–68.9%), with lower sensitivity in older adults (79.9%), increasing their risk of undertriage (43). When considering hospital destination, initial transport to Level I/II trauma centres lowered sensitivity to 73.4% and specificity to 63.9%, while final triage (including hospital-based adjustments) improved sensitivity to 82.6% but reduced specificity to 53.2% (43). Steps 1 and 2 (physiologic and anatomic criteria) were highly specific but lacked sensitivity, whereas steps 3 and 4 (mechanism of injury and special considerations) improved sensitivity but increased overtriage (43). The findings highlight the scheme as a moderately reliable tool, with age-related limitations suggesting a need for adjusted criteria, particularly for older adults, to improve accuracy (43).

#### 4.1.2 The Ohio trauma triage protocol

The Ohio Trauma Triage Protocol is a structured framework developed by the Ohio Department of Public Safety’s (ODPS) Trauma Committee to guide EMS personnel in identifying trauma patients who require specialised care (23). The protocol is established under the authority of the Ohio EMS Board, as defined in the Ohio Revised Code 4765.04, and mandates periodic review to ensure it minimises over-triage and under-triage while addressing the specific needs of paediatric and geriatric trauma patients (23). The triage criteria are divided into three components: physiological conditions, anatomical conditions, and additional considerations, as described by Werman and colleagues (23):

Physiological Conditions focus on identifying critically injured patients based on vital signs and level of consciousness. These include a GCS score of 13 or lower, loss of consciousness exceeding 5 min, deterioration in consciousness at the scene or during transport, and failure to localise pain. Respiratory distress indicators include a respiratory rate of fewer than 10 or more than 29 breaths per minute, the need for endotracheal intubation, or relief of tension pneumothorax. Circulatory shock is indicated by a SBP of less than 90 mmHg, an absent radial pulse with a present carotid pulse, or a heart rate exceeding 120 beats per minute in combination with evidence of haemorrhagic shock.

Anatomical Conditions highlight injuries that warrant immediate trauma centre transport. These include penetrating trauma to the head, neck, or torso, and significant penetrating injuries to the extremities proximal to the knee or elbow with neurovascular compromise. Other serious injuries include visible crush injuries, abdominal tenderness or distention, pelvic fractures, flail chest, and spinal cord injuries. Additionally, extremity injuries such as amputations proximal to the wrist or ankle, fractures of two or more proximal long bones, and neurovascular compromise are considered high risk. Burns covering more than 10% of total body surface area or involving critical areas such as the face, feet, hands, genitalia, or airway also necessitate specialised care.

Additional Considerations include the mechanism of injury and special factors that may influence patient outcomes. EMS personnel are trained to integrate these considerations into their decision-making, ensuring that trauma patients receive timely and appropriate care based on the severity of their injuries. The Ohio Trauma Triage Protocol serves as a critical decision-making tool for EMS providers, ensuring that patients receive the appropriate level of trauma care while optimising resource allocation within the healthcare system (23).

Ichwan and colleagues evaluated the reliability of the Ohio Trauma Triage Protocol, particularly its geriatric-specific criteria introduced in 2009, compared to the standard adult triage criteria in identifying older adults needing trauma centre care (22). Using data from 101,577 injured patients (33% geriatric), they found that the geriatric criteria significantly improved sensitivity for older adults (93%; 95% CI: 92–93%) compared to the adult criteria (61%; 95% CI: 60–62%). However, this improvement came with a decrease in specificity from 61% (adult criteria) to 49% (geriatric criteria). The geriatric criteria performed similarly to the adult criteria in younger adults (sensitivity: 87%, specificity: 44%), suggesting that age-adjusted triage improves case identification for older adults while modestly increasing overtriage (22).

The study also assessed secondary outcomes such as ICU admission, in-hospital mortality, and need for surgery within 48 h (22). Across these measures, the geriatric criteria consistently demonstrated higher sensitivity, ensuring more older adults with severe injuries were correctly identified (22). However, specificity was reduced, leading to an increased number of patients being triaged to trauma centres unnecessarily. Despite this trade-off, the findings highlight that standard adult EMS triage guidelines are insufficient for older adults, and geriatric-specific triage criteria significantly reduce undertriage in this population (22). The study supports age-adjusted modifications to triage protocols to improve accuracy and reduce the risk of missed injuries in older patients (22).

### 4.2 Challenges in developing geriatric-specific trauma triage tools

Currently, trauma triage tools do not effectively identify major trauma in older adults (22). Factors such as physiological criteria (21, 24), comorbidities (25), injury pattern, and mechanism (17) may affect the capacity of triage tools to meet specific criteria for older trauma patients (11). Triage tools also consider the distance between the patient and the MTC to determine whether they should be transported there (21, 25). Given these factors, it may be beneficial to develop geriatric-specific trauma triage tools. Researchers are working towards formulating such guidelines to meet the needs of older trauma patients, as well as meeting the recommendation that all trauma centres should adopt geriatric-specific guidelines (11, 24).

Efforts have been made to refine trauma triage tools for older patients (11). For instance, by incorporating an age criterion (> 55 years) as a mandatory element, one additional patient with severe injuries (Injury Severity Score, ISS > 15) was identified for every 60–65 patients transported to MTCs with less severe injuries (26). Meanwhile, altering the SBP threshold from < 90 mm Hg to < 110 mm Hg reduced under-triage by 4%, although over-triage increased by 4% (27). For older trauma patients with an SBP < 110 mm Hg, the risk of death is similar to those with an SBP > 90 mm Hg, hence this criterion should warrant direct transportation to an MTC (27). Additionally, using a GCS score of ≤ 14 rather than ≤ 13 for patients aged 70 and older increased the sensitivity of the triage tool from 50.7 to 59.2%, without compromising specificity when compared to its use in younger adults (20).

In some studies, specific trauma triage criteria have been developed for older adults. For example, an alternative triage tool in the US for trauma patients aged 65 and over showed greater sensitivity for detecting major trauma (ISS > 15) compared to adult triage guidelines (92% vs. 76%), though specificity was lower (42% vs. 78%) (40). Newgard and colleagues subsequently devised a clinical decision rule that accounts for geriatric physiology and comorbidities (28). While this rule had an overall sensitivity of 90%, specificity remained low (17%) for identifying older adults with ISS > 15 (28). In this study, anticoagulant use was not found to be a reliable predictor of high-risk patients, compared to other triage criteria (28).

Additionally, the Ohio EMS Board’s Trauma Committee developed a geriatric-specific triage guideline as an alternative to the Ohio trauma triage criteria for adults aged 16–59 years to improve identification of severely injured geriatric patients (22). Ichwan and colleagues clarified the key modifications: raising the SBP threshold from <90 mmHg to <100 mmHg and adjusting the GCS threshold from ≤13 to ≤14 for suspected traumatic brain injury (TBI). Additionally, the geriatric criteria consider a single proximal long bone fracture in a motor vehicle crash as a significant injury, unlike the standard criteria, which require fractures of two or more proximal long bones. Furthermore, the geriatric criteria incorporate specific mechanisms of injury that pose a greater risk to older adults. These include pedestrian collisions and falls from any height, including standing falls, if there is evidence of TBI—both of which were not explicitly covered in the standard protocol. Geriatric patients with injuries in two or more body regions are also prioritised for trauma centre transport. Ichwan and colleagues then compared the two guidelines and found that the geriatric-specific guideline increased sensitivity for patients aged ≥ 70 (93% vs. 61%) but reduced specificity (49% vs. 61%) compared to adult criteria (22). This tool performed comparably to adult triage tools in younger patients (sensitivity 93% vs. 87%, specificity 49% vs. 44%) (22).

### 4.3 Obstacles and recommendations for enhancing geriatric trauma triage

The following subsections demonstrates the key obstacles affecting the accuracy and effectiveness of prehospital trauma triage for older adults, highlighting challenges such as physiological differences, injury mechanisms, and undertriage risks. Additionally, recommendations are presented to enhance triage processes, including modifications to triage criteria, improved paramedic education, and the integration of frailty assessments to optimise trauma care for this vulnerable population.

#### 4.3.1 Obstacles and barriers to effective prehospital geriatric trauma triage

This section outlines key barriers affecting the accuracy and effectiveness of prehospital trauma triage for older adults. It explores challenges such as non-compliance with geriatric-specific triage tools, inaccuracies in triage leading to undertriage and overtriage, and the unique injury patterns in this population. Additionally, it examines physiological differences, injury mechanisms, and logistical challenges that contribute to triage errors. Addressing these barriers requires improved adherence to geriatric triage guidelines, enhanced paramedic training, and the integration of age-specific assessment criteria to ensure timely and appropriate care for older trauma patients.

##### 4.3.1.1 Compliance with geriatric-specific triage guidelines

The appropriate use and application of trauma triage tools are critical for ensuring that older trauma patients receive the necessary level of care. “Destination compliance” refers to the adherence to guidelines that ensure trauma patients meeting specific triage criteria are transported to a facility capable of providing the highest level of trauma care (11). Cox and colleagues also defined the destination compliance as ensuring that patients who meet prehospital trauma triage criteria reach the highest-level trauma service within a 30-min transport time (62). Paramedic judgement plays a vital role in prehospital triage decisions, but an optimal prehospital trauma triage system should aim to reduce the reliance on discretionary decision-making (62). A study reported that compliance with the US field triage guidelines for older adults is likely a nationwide issue, with significant implications for the quality of care and outcomes of geriatric trauma patients (29).

For instance, a patient with a GCS score of 8 and severe injuries who meets criteria for a MTC should ideally be transported to that facility (11). However, studies indicate that compliance with these guidelines diminishes with increasing patient age (11, 54, 62). In the United States, older adults who met triage criteria were only half as likely as younger adults to be transported to a designated MTC (62). Similarly, Ichwan and colleagues found the chance of being treated at a Level 1 trauma centre to be 89% less likely for a person aged 80 or older compared to a person aged <65 when both meet trauma triage criteria (22). Research in Australia revealed that 67% of older patients confirmed as major trauma cases were transported to an MTC, compared to 88% of younger patients (29, 62).

Several factors may contribute to this non-compliance, including a lack of triage sensitivity for older patients, age-based biases, patient or family preferences, and subjective decision-making by paramedics (26, 40, 62). Furthermore, potential biases related to age, ethnicity, and gender (particularly towards women) based on socio-economic factors need further exploration (25, 30). Surveys of paramedics also suggest that inadequate training, unfamiliarity with guidelines, ageism, and a perceived lack of respect in treating older patients can impact triage decisions (31). Research highlights how paramedic biases can lead to the under-triage of older adults due to assumptions about their recovery potential or frailty, resulting in non-compliance with destination protocols. For instance, a study found that such biases often deprioritise older patients (55), while another study reported a case where an older adult with severe injuries was transported to a lower-level facility rather than a MTC due to perceived comorbidities (56). In the United States, paramedics have been shown to rely on subjective judgments, with only 47% of older adults meeting geriatric-specific criteria initially transported to Level I/II trauma centres (14, 40).

Among the papers included in this review, the study by Cox and colleagues serves as a key example of research investigating compliance-related issues in triaging older trauma patients (62). Their study examined compliance with prehospital trauma triage destination criteria and its impact on older trauma patients’ outcomes in Victoria, Australia. Conducting a retrospective analysis of 25,042 trauma cases attended by Ambulance Victoria between 2007 and 2011, the study linked prehospital records with hospital data from the Victorian State Trauma Registry. The research specifically assessed whether age influenced adherence to triage protocols and the likelihood of older adults being transported to MTC. The findings revealed that while prehospital trauma triage criteria had a high sensitivity of 95.8%, under-triage rates increased significantly with age. Older trauma patients were 23.7% to 41.4% less likely to be transported to an MTS compared to younger patients. Additionally, for every year beyond 55 years of age, the mortality risk increased by 8%. These results suggest that paramedic discretion, comorbidities, and injury severity influenced triage decisions, often leading to non-compliance with destination criteria. The study concluded that optimising trauma systems for older patients is essential to reducing under-triage and improving outcomes, recommending enhanced geriatric-specific triage guidelines and further research into age-related triage challenges.

Older adults are frequently under-triaged and less likely to be transported to MTCs, with several possible explanations beyond what is explicitly studied (54). Due to high patient loads and resource constraints, paramedics may prioritise younger patients with better recovery prospects, often transporting older trauma patients to lower-level facilities, especially when their injuries are less apparent due to blunted physiological responses. Additionally, the complexity of geriatric assessments, compounded by pre-existing conditions and polypharmacy, may discourage thorough evaluations, resulting in expedited but non-compliant transport decisions. Logistical barriers, such as the distance of trauma centres from rural or suburban areas, further influence triage decisions, as transport time may take precedence over strict adherence to guidelines. Furthermore, shared decision-making with patients and their families can contribute to non-compliance when older adults prefer local hospitals over distant MTCs (66). While these explanations remain speculative, they align with known challenges in prehospital trauma triage, highlighting the need for further research to understand how operational pressures, biases, and patient preferences affect paramedic decision-making and compliance with geriatric-specific triage guidelines.

##### 4.3.1.2 Inaccuracy of prehospital triage for older adults and its consequences

Several factors contribute to the inaccuracy of prehospital triage for older adults. Firstly, low-energy mechanisms, such as falls from standing, are often the cause of injury in older adults, and these injuries may be overlooked by traditional triage systems (57). Secondly, age-related factors such as frailty, comorbidities, polypharmacy, and altered physiological responses to injury that may complicate the accuracy of triage tools for older patients (19). Assessing GCS scores in older adults may also be challenging due to existing physical or cognitive disabilities (70). Also, physiological variables like blood pressure may not be reliable indicators in older patients, as age-related changes alter their physiological norms (32). For instance, due to increased brain atrophy with age, older adults may maintain relatively high GCS scores even in the presence of intracranial bleeding (58).

Additionally, it is important to evaluate the consequences of triage tool use and the potential benefits of accessing specialist trauma care (33). Over-triage, which occurs when patients with minor injuries are unnecessarily transported to higher-level trauma centres, can result in wasted resources, higher costs, and inconvenience for both patients and their families due to increased distance from their homes (19). Conversely, under-triage failing to recognise severe injuries that can lead to patients being taken to facilities without appropriate trauma capabilities, thereby receiving suboptimal care (19). With the demographic shift leading to an increased number of serious injuries among older adults, prehospital triage systems have struggled, resulting in under-triage, higher mortality rates, and poorer recovery outcomes for older patients with major trauma (34, 59).

##### 4.3.1.3 Unique injury patterns and triage challenges in older trauma patients

Older trauma patients often present differently after injury compared to younger adults, which may limit the applicability of existing adult assessment and triage guidelines. Their injuries are typically caused by low-energy mechanisms, such as falls from less than 2 metres within the home environment (67). Older people are more susceptible to traumatic brain, thoracic, pelvic, and extremity injuries due to age-related physiological changes and increased fragility (67). Several unique factors also can negatively impact the physiological response and presentation of older adults following injury (60). These factors are primarily linked to age-related anatomical and physiological changes, comorbidities, and medication use (60). In older adults, altered physiological responses can affect key indicators such as heart rate, SBP, GCS, and respiratory rate; all of which are critical components of prehospital trauma triage criteria (67).

Additionally, older trauma patients are often injured in locations farther from trauma centres compared to younger adults, contributing to higher under-triage rates (11). A US study found that patients over 65 years old living in rural areas with limited trauma centre access were significantly more likely to be under-triaged in prehospital care (33). Distance has been shown to impact trauma triage decisions for patients aged 65 and older, with those residing more than 30 miles from a trauma centre having a 37% higher likelihood of under-triage (odds ratio [OR]: 1.37; 95% confidence interval [CI]: 1.15–1.40) compared to those living within 15 miles (30). Furthermore, the association between age and under-triage is even more pronounced among older adults with major trauma who live over 30 miles from a trauma centre, as the odds of under-triage increase by 64% for patients over 80 years old (OR: 1.64; 95% CI: 1.53–1.76) (30). Addressing these obstacles requires research on triage issues, targeted education, the incorporation of geriatric-focused case studies, and standardised triage protocols that prioritise objective physiological criteria to reduce biases in prehospital care and improve decision-making (54, 66).

#### 4.3.2 Evidence-based recommendations for optimising geriatric trauma triage

To enhance the triage process for older trauma patients, Alshibani and colleagues emphasised the need for guidance to assist paramedics in conducting appropriate assessments and making informed triage decisions, ultimately improving compliance in patient management (11). Along with adjusting and incorporating geriatric-specific triage criteria as discussed earlier, Alshibani and colleagues proposed a structured approach to optimising prehospital trauma triage by assessing physiological responses to injuries, evaluating the mechanism of injury, applying age-based triage cutoffs, considering comorbidities and medication use, integrating frailty assessment into triage decisions, and emphasising the importance of patient and carer involvement through shared decision-making (67).

##### 4.3.2.1 Assessment of physiological responses to trauma

It is important for paramedics to accurately assess important physiological responses for older patients’ injuries including signs of shock, altered mental status, and breathing distress (67). Research recommended that paramedics should apply a SBP threshold of <110 mmHg and a heart rate >90 bpm as criteria for the direct transport of older trauma patients to trauma centres (11, 60, 67). They should also be vigilant for early signs of shock, as older trauma patients may appear normotensive if only heart rate and SBP are considered. Any change in GCS should prompt direct transport to a trauma centre, along with a thorough collection of event details and medical history, as well as comprehensive primary and secondary assessments. Moreover, a respiratory rate <10 or >20 breaths per minute, or the need for ventilatory support, should indicate direct transport to a trauma centre. Paramedics must also be aware of altered physiological responses to hypoxia and hypercapnia in older adults and look for other signs of respiratory distress, including skin colour changes, grunting, nasal flaring, retractions, sweating, and abnormal body positioning (11, 60).

##### 4.3.2.2 Evaluation of the mechanism of injury

Low-level falls have become the leading cause of major trauma, yet prehospital trauma triage criteria remain insufficiently sensitive to these incidents (11). Paramedics’ judgement plays a role, as low-level falls are often perceived as causing only minor injuries (66, 67). However, the number of older adults experiencing major trauma due to such falls is rising (61). Adding “low-level falls” as a triage criterion may improve sensitivity but could reduce specificity and strain trauma centres (67). Incorporating frailty scores in prehospital triage could help identify high-risk older patients more effectively (67).

##### 4.3.2.3 Application of age-based triage cutoffs

Using age cutoffs in trauma triage has been proposed to improve the identification of high-risk older patients (67). However, setting 55 years as a mandatory prehospital triage criterion has been shown to increase overtriage, leading to unnecessary transport of non-severely injured patients to high-level trauma centres (11). In contrast, a study suggests that patients aged ≥70 years should be evaluated at trauma centres with an activated trauma team, as this could reduce in-hospital undertriage without significantly increasing overtriage (35). The issue of prehospital undertriage may begin as early as age 50, yet most studies have focused on mortality, despite other outcomes being more relevant for older adults (31). Given these complexities, paramedics should consider age alongside other triage criteria rather than relying solely on age cutoffs, as higher age has been associated with increased mortality regardless of injury severity (67).

##### 4.3.2.4 Consideration of comorbidities and medication use

Comorbidities are more common in older adults and have been identified as predictors of major trauma and major non-orthopaedic surgery when included in triage criteria. However, anticoagulant use alone was not found to be a reliable predictor (28). Certain medications, such as anticoagulants and antihypertensives, significantly increase the risk of intracranial haemorrhage after head trauma and severe fall-related injuries in older adults (36, 60). Polypharmacy is also a known risk factor for death and disability in this population (60). Incorporating anticoagulant and antiplatelet use into triage criteria for older patients with TBI has been shown to improve sensitivity in detecting intracranial haemorrhage, death, or the need for neurosurgery, with a modest trade-off in specificity (37). Therefore, paramedics should obtain a thorough and accurate medication history whenever possible to enhance triage decisions.

##### 4.3.2.5 Integration of frailty assessment

Frailty is a multidimensional syndrome reflecting a reduced ability to cope with stressors such as trauma, serious illnesses, or surgical interventions (55). It is a long-term condition linked to ageing and multiple comorbidities, reducing physiological reserve and resilience, making individuals more vulnerable to minor stressors (67). It is associated with the ageing process but is not universal among older adults (55). It increases with age, affecting around 10% of those over 65, with prevalence rising to 25–50% in those over 85 (46). Frailty is a critical determinant of clinical outcomes in older trauma patients, influencing their ability to recover from injuries and their risk of mortality (55).

Despite its significance, frailty assessment is often overlooked in trauma care pathways. A Canadian study revealed limited guidelines for paramedics in assessing frailty during routine trauma assessments (71). Similarly, a UK qualitative study found that paramedics lack adequate knowledge, training, and understanding of frailty and ageing-related changes, even though they are responsible for delivering optimal geriatric care (56). To address these gaps, recent Australian studies recommended integrating frailty assessments into all patient evaluations for those aged 65 and older (63, 64).

## 5 Possibility of integrating frailty assessment in prehospital trauma triage: feasibility, accuracy and clinical implications

Frailty assessment has emerged as a critical tool in identifying older trauma patients at high risk of adverse outcomes, offering potential improvements in prehospital trauma triage (47). Studies have demonstrated that frailty is a strong predictor of mortality, morbidity, and hospitalisation in older adults, including those with traumatic injuries (48, 74). Despite its clinical utility, frailty assessment tools have not yet been systematically integrated into prehospital trauma triage protocols (47). Incorporating frailty screening into the EMS could enhance clinical decision-making, optimise resource allocation, and improve trauma outcomes for older patients. Several frailty assessment tools have been evaluated for their feasibility and predictive accuracy in emergency department (ED) settings, including the Clinical Frailty Scale (CFS), Identification of Seniors at Risk (ISAR), and Programme on Research for Integrating Services for the Maintenance of Autonomy (PRISMA-7) (72) (Table 2).

**Table 2:** A summary of frailty assessment tools in emergency and trauma care: features, accuracy, and prehospital considerations.

Tool	Key features	Evidence of predictive accuracy	Strengths	Limitations / barriers to prehospital use
PRISMA-7 (Programme on Research for Integrating Services for the Maintenance of Autonomy)	7-item questionnaire Self-reported Screens for disability and comorbidity	AUC: 0.88 Sensitivity: 84%, Specificity: 78% Best at distinguishing pre-frail vs. frail (AUC: 0.71)	High diagnostic accuracy Short administration time Strong inter-rater reliability (*r* = 0.75) Effective in ED triage settings	Not yet validated for EMS use Dependent on patient response No digital tool for EMS application Requires further feasibility testing in prehospital settings
CFS (Clinical Frailty Scale)	9-point clinical judgement scale Based on functional status and comorbidities Requires trained rater	AUC: 0.83 Validated in trauma patients Strong predictor of 30-day mortality and adverse outcomes	Greater specificity than ISAR or PRISMA-7 Predicts mortality, delirium, and discharge needs Widely used in EDs across multiple countries	Moderate inter-rater reliability (*r* = 0.78) Requires clinical training Longer assessment time than PRISMA-7 Not routinely used in prehospital triage
ISAR (Identification of Seniors at Risk)	6-item screening tool Self-reported Focuses on prior hospital use, ADLs, memory	AUC: 0.78 Sensitivity: 95%, Specificity: 35% Highest sensitivity, but lowest specificity	Quick to administer High sensitivity Suitable for initial risk identification	Poor specificity → high false-positive rate Weakest diagnostic accuracy overall Reliability lower (*r* = 0.62) May overburden trauma centres if used without refinement

Among 265 patients screened, 58% were classified as frail based on a comprehensive geriatric assessment (CGA). PRISMA-7 demonstrated the highest diagnostic accuracy (AUC 0.88, 95% CI: 0.83–0.93), followed by CFS (AUC 0.83, 95% CI: 0.77–0.88) and ISAR (AUC 0.78, 95% CI: 0.71–0.84) (72). While PRISMA-7 was significantly more accurate than ISAR (*p* = 0.008), it was not statistically different from CFS (*p* = 0.15) (72). Additionally, PRISMA-7 was the most effective tool in differentiating pre-frail from frail individuals (AUC 0.71), reinforcing its suitability for use in ED triage settings (72).

Frailty screening has been increasingly studied in emergency care settings, with strong evidence supporting the CFS as a triage tool in ED (38, 49, 65, 75). Studies from Australia, Switzerland, the United Kingdom, and the United States have consistently shown that CFS accurately predicts hospital-related outcomes in older trauma patients (38, 49, 65, 75). A prospective study comparing frailty assessments in older trauma patients found CFS to be both feasible and accurate when compared to PRISMA-7 and the Trauma-Specific Frailty Index (50). Moreover, frailty has been identified as an independent predictor of 30-day mortality, inpatient delirium, and increased care needs at discharge (48, 51). These findings highlight the potential role of frailty screening in trauma risk stratification, particularly in EMS settings, where early identification of high-risk patients is essential.

The feasibility of frailty assessment in emergency settings depends on factors such as administration time, reliability, and predictive performance. O’Caoimh et al. reported that CFS, PRISMA-7, and ISAR all had relatively short administration times and demonstrated moderate to strong inter-rater reliability (ISAR: *r* = 0.62, CFS: *r* = 0.78, PRISMA-7: *r* = 0.75) (72). However, notable differences in sensitivity and specificity were observed. ISAR exhibited the highest sensitivity (95%) at its recommended cutoff but suffered from poor specificity (35%), leading to a high false-positive rate. PRISMA-7 provided a more balanced approach, with sensitivity (84%) and specificity (78%), making it the most reliable tool for frailty identification in ED triage. In contrast, CFS demonstrated greater specificity but lower sensitivity, reflecting its reliance on trained raters rather than self-reported data. Given its superior diagnostic accuracy and ease of administration, PRISMA-7 has been recommended as the most effective tool for frailty screening in ED settings, with potential applications in prehospital trauma triage (72).

Despite strong evidence supporting the predictive value of frailty assessments, their integration into prehospital trauma triage remains limited, with no routine use in EMS settings. Barriers such as the absence of standardised protocols, time constraints, variability in paramedic training, and limited resources hinder their implementation (66, 68). The need for rapid decision-making in prehospital care further challenges the feasibility of comprehensive frailty screening, while the lack of digital tools adds to the difficulty of manual assessments. Although tools like CFS, ISAR, and PRISMA-7 have demonstrated high predictive accuracy in hospital settings, future research should assess their feasibility and clinical impact in prehospital trauma care, as integrating frailty screening into triage protocols could enhance risk stratification, reduce undertriage, and improve outcomes for older trauma patients.

## 6 The trauma centre access and benefits of specialist trauma care for older trauma adults

There is also conflicting evidence regarding the impact of trauma centre access on survival and recovery outcomes for older trauma patients (11). Some studies have shown that under-triaging older patients is linked to higher rates of death, disability, and complications (17, 24). However, trauma centres are also associated with increased healthcare costs and longer hospital stays compared to non-trauma facilities (11). Furthermore, major trauma is often defined by an ISS greater than 15 (19). Not all patients with high ISS will benefit from specialist care, especially those with unsurvivable injuries or significant comorbidities, which may render bypassing local support systems unnecessary (19). Patients and their families may also prefer to receive treatment closer to home, even if that means a lower likelihood of recovery (19).

Evidence regarding the benefit of specialist care for older people with head injuries is limited. Some studies have suggested that older adults with intracranial injuries may not benefit from neurosurgical interventions, and transferring them to an MTC may not provide additional value (52, 53). Those with serious head injuries deemed unsuitable for neurosurgery might be better served by being treated at a local hospital where they can receive rehabilitation or end-of-life care near their homes (53). A recent review highlighted the increasing prevalence of TBIs among older adults, yet noted a scarcity of clinical guidelines for their acute and long-term management (39). This lack of evidence-based guidelines has led some centres to impose strict age cut-offs for treating severe head injuries in older adults (69). Meanwhile, others admit older adults with any head injury to neuro-intensive care units for serial neurological monitoring and head CT scans—an approach that may be excessively conservative (39). Prognostic models and evidence-based treatment guidelines are needed to determine which older adults with head injuries would benefit most from aggressive versus conservative treatment approaches (39).

## 7 Conclusion

This narrative review highlights the critical need for tailored approaches to prehospital trauma care for older adults, given the unique challenges posed by the ageing population. Existing trauma triage tools, originally designed for younger patients, often fail to account for age-related physiological changes, frailty, and polypharmacy, leading to significant rates of under-triage and over-triage. While advancements such as age-specific criteria, frailty assessments, and adjusted physiological thresholds have shown promise in improving the accuracy of triage tools, their widespread implementation remains hindered by barriers such as inconsistent guidelines, insufficient training, and age-based biases. Despite these insights, significant gaps in research persist. There is a lack of robust, large-scale prospective studies evaluating the real-world effectiveness of modified triage criteria for older adults. The feasibility of integrating frailty assessments into prehospital triage also remains unclear, with limited research on how paramedics can efficiently incorporate such assessments into their decision-making processes. Furthermore, while evidence suggests that trauma centre access improves outcomes for older adults, further studies are needed to determine which patients benefit most from specialist care, ensuring that triage modifications do not result in unnecessary resource utilisation. Additionally, research on the long-term functional outcomes of older trauma patients following different triage decisions is scarce, making it difficult to assess the broader impact of current prehospital triage strategies.

The review underscores the importance of developing and adopting standardised geriatric-specific triage protocols to ensure that older adults receive appropriate and timely trauma care. It also highlights the need for further research to refine triage tools, incorporate frailty assessments, and address gaps in paramedic training and decision-making processes. By improving access to specialist trauma centres and integrating evidence-based practices, prehospital care systems can better meet the needs of this vulnerable population. Moving forward, expanding the role of EMS to include frailty assessments, enhancing paramedic education, and addressing biases in prehospital care are essential steps. Such efforts will not only improve patient outcomes but also optimise resource utilisation and support a more equitable trauma care system for older adults. Future research should focus on evaluating the clinical impact of geriatric-specific triage tools, exploring the role of digital decision-support technologies for EMS personnel, and investigating how prehospital triage decisions influence long-term recovery and quality of life in older trauma patients. Addressing these gaps will enhance the precision of prehospital triage, optimise resource allocation, and ultimately improve outcomes for older adults experiencing major trauma. This work aims to inform future research and policy development, contributing to the advancement of geriatric trauma care practices worldwide.
